# High glucose induces human endothelial dysfunction through an Axl-dependent mechanism

**DOI:** 10.1186/1475-2840-13-53

**Published:** 2014-02-26

**Authors:** Chien-Hsing Lee, Yi-Shing Shieh, Fone-Ching Hsiao, Feng-Chih Kuo, Chih-Yuan Lin, Chang-Hsun Hsieh, Yi-Jen Hung

**Affiliations:** 1 Division of Endocrinology and Metabolism, Department of Internal Medicine, Tri-Service General Hospital, National Defense Medical Center, #325, Section 2, Cheng-Gong Rd., Nei-Hu, Taipei, Taiwan; 2 School of Dentistry, National Defense Medical Center, Taipei, Taiwan; 3 Department of Oral Diagnosis and Pathology, Tri-Service General Hospital, Taipei, Taiwan; 4 Division of Cardiovascular Surgery, Department of Surgery, Tri-Service General Hospital, National Defense Medical Center, Taipei, Taiwan

**Keywords:** Diabetes, Endothelial cell, Gas6/Axl

## Abstract

**Background:**

The receptor tyrosine kinase Axl and its ligand growth arrest-specific protein 6 (Gas6) are involved in the diabetic vascular disease. The aim of this study was to explore the role of Gas6/Axl system in high glucose (HG)-induced endothelial dysfunction.

**Methods:**

We investigated the effect of various glucose concentrations on Axl signaling in human microvascular endothelial cells (HMEC-1 s).

**Results:**

Human plasma Gas6 value inversely correlated with glucose status, endothelial markers. HG decreased Gas6/Axl expression and increased intercellular adhesion molecule-1 (ICAM-1) and vascular cell adhesion molecule-1 (VCAM-1) expression in HMEC-1 s. HG significantly decreased HMEC-1 s cell viability and tube formation and promoted monocyte-EC adhesion. Down-regulation of Akt phosphorylation was found in HG culture. Axl transfection significantly reversed HG-induced Akt phosphorylation, VCAM-1 expression and endothelial dysfunction. We also found additive changes in Axl-shRNA-infected HMEC-1 cells in HG culture. Furthermore, Axl overexpression in HMEC-1 s significantly reversed HG-induced vascular endothelial growth factor (VEGF) and VEGF receptor 2 (VEGFR2) expression. In addition, significantly lower Axl and VEGFR2 expression in arteries were found in diabetic patients as compared with non-diabetic patients.

**Conclusions:**

This study demonstrates that HG can alter Gas6/Axl signaling and may through Akt and VEGF/VEGFR2 downstream molecules and suggests that Gas6/Axl may involve in HG-induced EC dysfunction.

## Background

Diabetes mellitus (DM) is a common metabolic disease with a high and increasing prevalence worldwide. The estimated number of patients afflicted with diabetes, presently about 170 million people, is expected to increase and reach a pandemic rate of 366 million by the year 2030 [1]. The diabetic condition is characterized by and is responsible for alterations in micro- and macro-vascular beds, by inducing changes in neovascular mechanisms and impairing vascular homeostasis [2,3]. Dysfunction of the vascular endothelium is regarded as an important factor in the pathogenesis of micro- and macro-angiopathy [4,5] and endothelial function has gained increasing attention in the study of vascular disease. Endothelial cell (EC) injury and proliferative dysfunction are considered to be the initial events in the development of atherosclerosis, postangioplasty restenosis, plaque erosion, and thromboembolism, which are contributors to macro-vascular complications [5]. Inhibition of the vascular endothelial growth factor (VEGF)–VEGF receptor 2 (VEGFR2) signaling axis may play a role in EC dysfunction and serious vascular complications associated with diabetes [6]. However, the mechanisms by which diabetes-induced EC dysfunction occurs remain poorly identified.

Growth arrest-specific protein 6 (Gas6) belongs to the family of vitamin K-dependent coagulation proteins and is recognized as a growth factor-like molecule, as it interacts with receptor tyrosine kinases of the TAM (Tyro-3, Axl, Mer) family [7]. The Gas6/TAM system regulates an intriguing mix of processes, including cell survival and proliferation, cell adhesion and migration, blood clot stabilization, and inflammatory cytokine release. Hence, the role of the Gas6/TAM system has been found to be important in injury, repair, inflammation, hemostasis, autoimmune disease, vascular systems, and cancer [8]. Over the years, numerous studies have identified the pivotal role Gas6 plays in vascular biology and diseases [9,10]. Gas6 and its receptors are involved in the pathogenesis of neointima formation, vasculitis and atherosclerosis [11-14].

Recently, several reports revealed that the Gas6/TAM system was involved in the pathogenesis of diabetic renal and vascular disease. Expression of Gas6/TAM was increased in the glomerulus of diabetic rats, which led to mesangial and glomerular hypertrophy [15]. In vascular smooth muscle cells, Gas6/Axl signaling increased cell survival in the presence of low glucose (LG) and increased cell migration in the presence of high glucose (HG) [16]. Our report also elucidates plasma Gas6 levels are associated with altered glucose tolerance, inflammation, and endothelial dysfunction. Plasma Gas6 concentration may represent an independent risk factor of type 2 diabetes, and a potential surrogate marker of inflammation and endothelial dysfunction [17]. These studies indicate that Gas6/TAM likely represents an important pathogenic mechanism for renal and cardiovascular complications associated with diabetes.

The sequence and nature of the antiapoptotic and angiogenesis events resulting from Gas6/Axl interactions have been most extensively studied in murine NIH-3 T3 fibroblasts and many different types of cancer [18-21]. In this system, Gas6/Axl interactions activate Akt through phosphorylation. Akt itself is a serine-threonine kinase that has been shown to be a key intracellular regulator of cellular survival. Activation of Akt leads to downstream signaling events, including those associated with the mitochondrial regulation of apoptosis and angiogenesis [15]. Interestingly, the Gas6/Axl pathway can interact with other growth factor signals, as has been shown for VEGFR2 in EC morphogenesis [22]. However, the plausible role of Gas6/Axl molecules and its downstream signaling in the context of hyperglycemia and endothelial dysfunction has never been explored. In the present study, we demonstrate for the first time that HG causes Gas6/Axl/Akt-dependent down regulation of VEGF and VEGFR2 expression. Furthermore, a decrease in cell viability and angiogenesis and increased vascular cell adhesion molecule-1 (VCAM-1) production and induction of monocyte-endothelial cell adhesion in human microvascular endothelial cells (HMEC-1) are observed.

## Methods

### Cell culture

Immortalized HMEC-1 were maintained in medium MCDB-131 (Sigma) supplemented with 15% FBS (GIBCO, Carlsbad, CA), 10 ng/ml EGF (R&D Systems, Minneapolis, MN), 1 μg/ml dsdahydrocortisone (Sigma, St. Louis, MO) and 10 mM L-glutamin (GIBCO, Carlsbad, CA) at 37 in a humidified 5% CO2 incubator. For high glucose, cells were grown in 20 or 40 mmol/L glucose and controls received 20 or 40 mmol/L mannitol or 5 mmol/L glucose. Human embryonic kidney cell line HEK293T were maintained in medium DMEM (GIBCO, Carlsbad, CA) supplemented with 10% FBS.

### Transfection

Axl knockdown plasmids (TRCN575-sequense 5′CGAAATCCTCTATGTCAACAT and TRCN576-sequense 5′GCTGTGAAGACGATGAAGATT) were purchased from Academia Sinica RNAi core, Taiwan. Axl overexpression plasmid was constructed in pCMV-Tag2A (pCMV-Tag2A/Axl(s)). Axl cDNA was amplified by RT-PCR with sense 5′-CCCAAGCTTGGAAAGTTTGGCACCCATG-3′ and antisense 5′-CCCAA GCTTGGTTGTCTCAGGCACCATC-3′. HMEC-1 cells were transfected with knockdown or overexpression plasmid using lipofectamine™ 2000 transfection reagent according to the manufacturer’s instructions (Invitrogen). Knockdown assay was performed with lent virus system and the protein expression was confirmed by western blot of Axl.

### RNA extraction and RT-PCR

Cryogenic conditions were used for mRNA extraction, which was performed following the protocol described for the commercial reactive TripureR (Roche Applied Science, Indianapolis, IN). The total RNA was extracted, purified, and converted to cDNA using oligo d(T)^12–18^ primer to preserve the relative mRNA profile and produce a template suitable for the PCR. In the PCR step, 50 pmol each of sense and antisense primers were used. The primer sequences were the following: sense 5′-CCACATCGCTCAGACACCAT-3′and antisense 5′-TGACCAGGCGCCCAAT A-3′ for GAPDH and sense 5′-TGTCCTAGCCTGTGTGTCAGTGA-3′ and antisense 5′-GGACCCTGGTGGCTGTGCCCCCTGTC-3′ for Axl and sense 5′-GCATCA ACAAGTATGGGTCTCC′ and antisense 5′-CCCAAGTCCATCTCACTATTTAC AG-3′ for Gas6. Standard PCR amplification conditions were applied, which consisted of a hot start at 94°C for 5 minutes, followed by 94°C for 30 seconds, 55°C for 30 seconds, and 72°C for 1 minute for 30 cycles, with a final amplification at 72°C for 10 minutes. Each measurement was performed in duplicate, and the threshold cycle value was determined for each amplification curve. The geometric mean of the GAPDH endogenous expression was used for the normalisation of the expression.

### Western blot

Western blotting was performed with a SDS-PAGE electrophoresis system. Briefly, protein samples were re-suspended in sample buffer (containing 10% β-mercaptoethanol) and electrophoresed on a 10% SDS-PAGE. Protein were transferred electrophoretically onto a polyvinylidene fluoride (PVDF) membrane and nonspecific binding sites blocked by immersion of membrane into 5% - milk. The membrane was incubated with primary antibodies against Axl, Gas6, ICAM-1, VCAM-1 and VEGFR2 (Santa Cruz, CA, USA), p-Akt, Akt, p-44/42 MAPK, 44/42 MAPK (both from Cell Signaling Technology, Danvers, MA) and GAPDH antibody (Novus Biologicals, Littleton, CO) was used as loading control. A horseradish peroxidase-conjugated mouse anti-goat, goat anti-mouse, goat anti-rabbit antibody (purchased from Jackson, USA) was then added as secondary antibody. Protein was detected by Luminata Classico Western HRP Substrate (Millipore, Bedford, MA). Semi-quantification of relative protein expression was analyzed with Image J.

### Cell viability assay

The influence of each concentration of HG to HMEC-1 was assessed by MTT assay. HMEC-1 were seeded at a density of 5,000 and 10,000 cells/well in 96-well plates and allowed to attach. Cells were treated with HG medium (serum free) for 0, 1, 3, 5, and 7 days with MTT (Usb) then added and survival determined. The data reported represent the mean ± SD of each experiments performed with 3 replicates tested.

### Migration

HMEC-1 were seeded at a density 3 × 10^6^ cells/well in 6 well plates and allowed to attach. After making a straight scratch with a sterile 200 pipette tip, a wound was simulated and cells were treated with high glucose medium (serum free). Both wound edges were monitored and recorded after 2, 4, 8, 12 h using the fluorescence microscopy (4 × magnification, OLYMPUS CKX41 coupled with camera).

### ELISA

The VEGF released by HMEC-1 in the culture medium was quantified using a Human VEGF ELISA kit (R&D Systems, Minneapolis, MN) according to the manufacturer’s instructions. The VEGF was measured in the culture medium of the cells exposed to all experimental conditions.

### Cell adhesion test

HMEC-1 cells (1 × 10^6^ cells/ml) were cultured in normoglycemic (NG, 5 mmol/L glucose) and hyperglycemic (HG, 40 mmol/L glucose) of 6-well culture plate, Incubate the cells for 24 h in CO_2_ incubator. The fluorescence labeling of THP-1 with calcein-AM, incubate the cells (1 × 10^7^ cells/ml) with 5 μ calcein-AM in RPMI 1640 for 30 min at 37°C in CO_2_ incubator. The cells were washed three times with PBS to remove excess dye and resuspended in phenol red-free RPMI 1640 (with 10% FBS) at a density of 1 × 10^6^ cells/ml. After high glucose treat the HMEC-1 cell co-cultured with calcein-AM labeled cells (1 × 10^6^/ml, well) in CO_2_ incubator at 37°C for 1 h. The HMEC-1 cell were washed four times with PBS to remove the non-adherent calcein-AM labeled cells and replaced with 1.0 ml of PBS. The fluorescence of each well was measured using a fluorescence microscopy with excitation and emission wavelengths of 480 nm and 530 nm, respectively.

### Tube formation

BD Matrigel™ Basement Membrane Matrix (BD Biosciences, San Jose, CA) was thawed overnight at 4°C and mixed to homogeneity with condition medium without serum. Culture plates (24 well) were coated with 0.3 mL of matrigel and allowed to gelatinize at 37°C for 3 hours. After centrifugation, HMEC-1 cells (1 × 10^5^) were resuspend in high glucose medium and seeded in matrigel. The EC-directed tube formation was monitored and recorded after 16–18 h of incubation using the fluorescence microscopy (4 × magnification, OLYMPUS CKX41 coupled with camera). The average length of tubes formed was counted in five different fields in each of the three independent repeats and quantified by Image J.

### Human samples

A total of 300 adults were recruited from the outpatient clinics of Tri-Service General Hospital, Taipei, Taiwan. Criteria for inclusion and exclusion into this study and the analytic methods of clinical variables in this study were the same as our previous study [17]. In addition, ten patients undergoing coronary artery bypass grafting (CABG) for documented coronary artery disease were recruited for the present study. All patients undergoing CABG surgery enrolled in this study were hemodynamic stable patients and the operation was done in an elective fashion. Discarded human left internal mammary artery (LIMA) tissues were obtained from patients undergoing CABG. The institutional review board of the Tri-Service General Hospital approved the protocol and all subjects provided written informed consent.

### Frozen section and immunohistochemistry

Discarded human IMA were taken from the operation room and placed immediately at -80°C. The snap frozen tissues were embedded with OCT (Surgipath, #01480, Leica Biosystems, Nussloch, Germany) and equilibrate at -20°C for frozen sectioning to 5 μm. Sections were air dried for 5 min and washed to remove OCT. For immunohistochemical staining, each section was blocked with blocking solution for 1 h and incubated for 15 minutes in 3% H2O2 diluted in methanol, with complete washing of sections between each steps. Specimens of LIMA were stained with primary antibodies: rabbit polyclonal to Axl (Abcam, #ab37861, Cambridge, UK,) and rabbit polyclonal to VEGFR2 (#55B11, Cell Signaling Technology, Danvers, MA) diluted in Dako diluent (Dako, #s3022, Dako Cytomation, Glostrup, Sweden) for 1 h at room temperature followed by detection with the Dako REAL EnVision system (Dako, #K5007, Dako Diagnostics, Dublin, Ireland) and mounted under cover slips.

### Protein isolation from frozen/OCT-embedded samples

After excess OCT was washed with ddH2O and 1X PBS, each tissue section was moved to a 2 ml tube containing ceramic beads (2.8 mm, bertin technologies). Lysis buffer (50 mM Tris–HCl [pH7.4], 150 mM NaCl, 2 mM EDTA, 1% NP-40, 0.1% SDS, protease inhibitor) was added, and the sample was homogenized by vortexing and grinding in a homogenizer (Precellys®24, bertin technologies, France). The sample was kept on ice for 30 min to complete the lysis reaction and centrifuged (4°C, 13000 g, 15 minutes) for supernatant collection. Collected sample was stored at -80°C.

### Statistical analysis

Descriptive results of continuous variables were expressed as means ± standard error of the mean (SE). We used unpaired *t* test and ANOVA test for comparisons of quantitative variables. Relationships between variables were tested using Spearman rank-order correlations and partial correlation analysis after adjusting for age. Proliferation data are means ± SE. Immunoblot data are means ± SD of band intensity relative to control. All experiments were repeated for *n* = 3–6. Groups are analyzed for differences by one-way ANOVA followed by Tukey's test. Significance was considered as being accepted at *P* < 0.05. All the statistical analyses were performed using the program SPSS (Chicago, Illinois, USA; version 13.0).

## Results

### Human plasma Gas6 protein concentrations are associated with glucose tolerance and endothelial dysfunction

**Figure 1:**
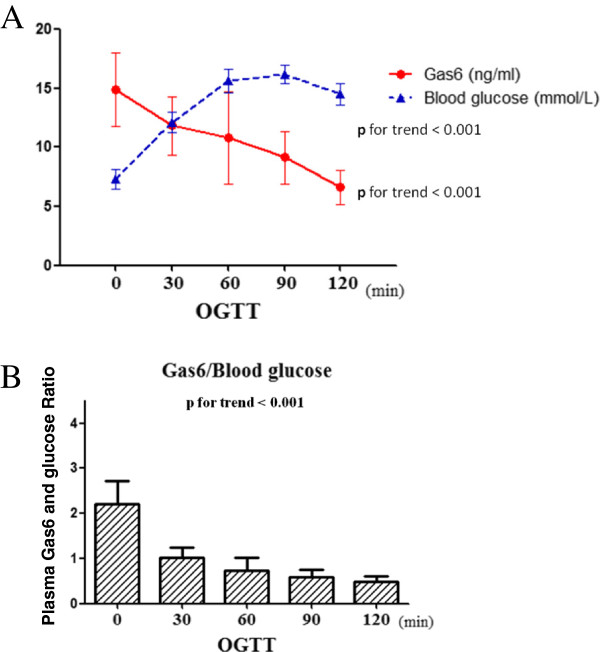
**Human plasma Gas6 protein concentrations are associated with corresponding glucose levels during OGTT.** A total of 104 type 2 diabetic patients were collected and received OGTT. **(A)** The plasma Gas6 value was significantly inversely correlated with glucose levels during OGTT (p for trend < 0.001). **(B)** The plasma Gas6 and glucose ratio showed significant reduced during OGTT (p for trend < 0.001). Lower plasma levels of Gas6 tended to be associated with higher plasma glucose levels.

In our previous report [17], we demonstrated that plasma Gas6 concentrations were significantly lower among patients with type 2 diabetes compared with subjects with NGT as illustrated in Table 1. In all subjects as a whole, the plasma Gas6 value was significantly and inversely correlated with glucose status and endothelial dysfunction markers (Table 2). In addition, we evaluated the association between plasma Gas6 protein levels and corresponding glucose values under acute glucose challenge using oral glucose tolerance test (OGTT) in 104 type 2 diabetic patients. In Figure 1, the plasma Gas6 value was significantly and inversely correlated with glucose levels during OGTT (p for trend < 0.001).

**Table 1:** Anthropometric and biochemical variables among different glucose tolerance subjects

	**NGT**	**IGT**	**Type 2 Diabetes**	* ***P *** **value**
	**(n = 100)**	**(n = 96)**	**(n = 104)**	
Age (years)	51.3 ± 1.46	55.1 ± 1.35	53.8 ± 1.28	0.032
Sex (M/F)	45/55	36/60	59/45	0.005
Blood pressure (mmHg)				
Systolic	119.4 ± 1.56	124.7 ± 1.69	126.8 ± 1.78	0.005^‡^
Diastolic	75.4 ± 0.82	77.6 ± 1.24	83.2 ± 1.13	0.006^‡^
OGTT glucose (mmol/L)				
Fasting glucose	5.03 ± 0.08	5.48 ± 0.12	8.34 ± 0.38	<0.001^‡^
2 h glucose	6.30 ± 0.06	10.13 ± 0.11	17.1 ± 0.36	<0.001^‡^
HbA_1C_ (%)	5.5 ± 0.02	6.1 ± 0.06	8.4 ± 0.13	<0.001^‡^
HOMA-IR^**§**^	2.02 ± 0.18	3.42 ± 0.22	4.89 ± 0.28	<0.001^‡^
Endothelial dysfunction markers				
E-selectin (ng/ml)	44.8 ± 1.53	48.5 ± 1.82	62.3 ± 2.32	<0.001^‡^
VCAM-1 (ng/ml)	518.2 ± 32.12	514.2 ± 36.81	668.4 ± 39.23	0.005^‡^
ICAM-1 (ng/ml)	245.2 ± 8.78	246.2 ± 8.61	295.2 ± 9.52	<0.001^‡^
Gas6 (ng/ml)^**§**^	15.2 ± 0.42	13.5 ± 0.48	11.2 ± 0.31	0.002^‡^

*assessed by one-way ANOVA, data shown as mean ± standard error mean.

^**§**^The logarithms of these variables were used for the analysis.

^**‡**^NGT vs. type 2 diabetes. All assessed by post-hoc LSD test.

NGT, normal glucose tolerance; IGT, impaired glucose tolerance; OGTT, oral glucose tolerance test; ICAM-1, intercellular adhesion molecule 1; VCAM-1, vascular cell adhesion molecule 1.

**Table 2:** Age-adjusted Spearman partial correlation coefficients between plasma Gas6 concentration and biochemical variables

	Spearman partial correlation coefficient*	
	**All (n = 300)**	
	**r**	***P***
OGTT glucose (mmol/L)		
Fasting glucose	-0.186	0.002
2 h glucose	-0.162	0.007
HbA_1c_ (%)	-0.158	0.029
E-selectin (ng/ml)	-0.161	0.008
VCAM-1 (ng/ml)	-0.282	<0.001

*Corrected for age OGTT, oral glucose tolerance test; VCAM-1, vascular cell adhesion molecule 1.

### HG concentrations and Gas6/Axl, ICAM-1 and VCAM-1 expression in HMEC-1 s

**Figure 2:**
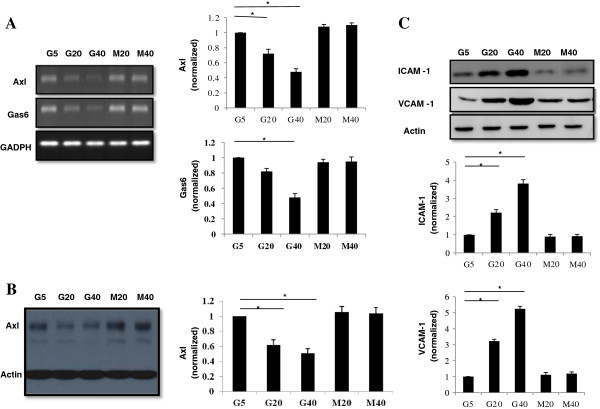
**HG concentrations and Gas6/Axl, ICAM-1 and VCAM-1 expression in HMEC-1 s.** HMEC-1 s were cultured in 5, 20 and 40 mM glucose and equimolar concentrations of mannitol for 24 h. **(A,B)** Gas6/Axl mRNA and Axl proteins expression were decreased by HG. **(C)** The expressions of ICAM-1 and VCAM-1 proteins were increased with a glucose dose-dependent manner. HMEC-1 s grown in mannitol did not change in Gas6/Axl, ICAM-1 and VCAM-1 expression.

To determine whether HG affects Gas6/Axl, ICAM-1 and VCAM-1 expression in ECs, HMEC-1 s were cultured in 5, 20 and 40 mM glucose and equimolar concentrations of mannitol for 24 h. As compared to the HMEC-1 s in normal glucose, Gas6/Axl mRNA and Axl proteins expression were decreased by HG (Figure 2A,B). However, the expression of endothelial functional markers, ICAM-1 and VCAM-1 proteins, were significantly increased at 20 and 40 mM glucose compared with 5 mM glucose in HMEC-1 s. (Figure 2C). HMEC-1 s grown in mannitol did not change in Gas6/Axl, ICAM-1 and VCAM-1 expression (Figure 2A-C). Therefore, the effect of D-glucose on these proteins expression was not secondary to osmotic load. These data showed that HG leads to a decrease in Gas6/Axl mRNA and protein level in HMEC-1 s, which likely contributes to endothelial dysfunction with increased ICAM-1 and VCAM-1 proteins expression.

### HG concentrations and HMEC-1 cell viability, adhesion, tubule formation and migration

**Figure 3:**
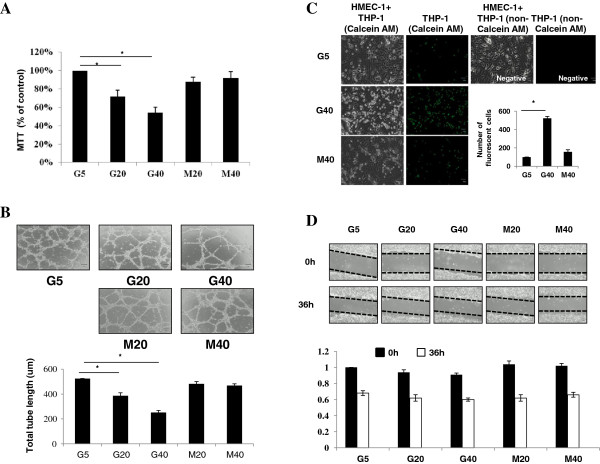
**HG concentrations and HMEC-1 cell viability, adhesion, tubule formation and migration. (A,B)** HG significantly decreased HMEC-1 s survival and tube formation with dose-dependent manner. **(C,D)** HG promoted significant monocyte-EC adhesion but did not influence HMEC-1 cell migration.

As summarized in Figure 3, HMEC-1 s were cultured in HG and mannitol for endothelial function tests. In Figure 3A, we measured the proportion of live HMEC-1 cells remaining after 72 h in the 5, 20 and 40 mM glucose and equimolar concentrations of mannitol using the MTT assay. HG significantly decreased HMEC-1 s survival with dose-dependent but not mannitol. To investigate the role of HG in HMEC-1 cell angiogenesis, we preformed the tube formation assay. Figure 3B, illustrates that HG elicited a dose-dependent decrease in tube formation. Significant decreased tube formations were evident at 20 and 40 mM glucose but not mannitol. We then evaluated the effect of HG in THP-1 (Human monocyte cell line) adhesion to HMEC-1 by using the monocyte-EC adhesion assay. As indicated by adhesion rate, HG promoted significant monocyte-EC adhesion (Figure 3C). However, HG and mannitol did not influence HMEC-1 cell migration when cells were maintained in migration assay for 36 h (Figure 3D).

### HG concentrations and Akt expression in HMEC-1 s

**Figure 4:**
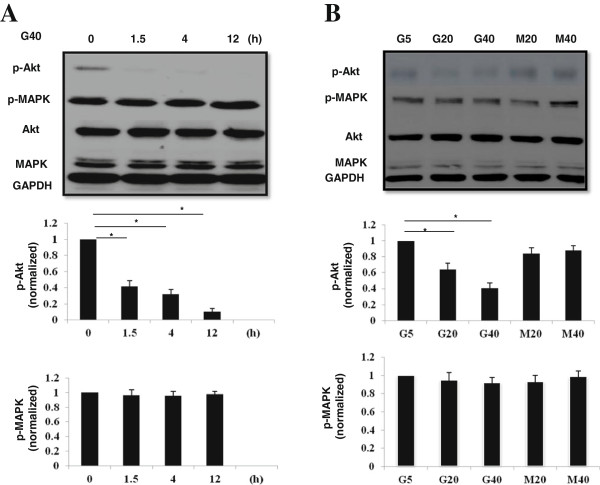
**HG concentrations and Akt expression in HMEC-1 s. (A)** In a time dependent manner, decreased Akt phosphorylation but no changes in MAP kinase were shown 0.5 to 12 hours after HG (40 mM D-glucose) treatment. **(B)** Down-regulation of Akt phosphorylation was noted after HG treatment with a dose dependent manner, whereas no change was observed for MAP kinase phosphorylation.

In order to further evaluate the involvement of downstream signaling in HG-triggered HMEC-1 s, Akt and MAP kinase activities were examined by determining the phosphorylated status of Akt and MAP kinase using the anti–phospho-Akt and anti–phospho-MAP kinase antibody. In a time dependent manner, decreased Akt phosphorylation but no changes in MAP kinase were shown 0.5 to 12 hours after HG (40 mM D-glucose) treatment (Figure 4A). In addition, down-regulation of Akt phosphorylation was noted after HG treatment with a dose dependent manner, whereas no change was observed for MAP kinase phosphorylation (Figure 4B). These data would elucidate the major downstream signaling that involved in HG-induced HMEC-1 s dysfunction is Akt signaling.

### HG concentrations and Axl transfection in HMEC-1 s

**Figure 5:**
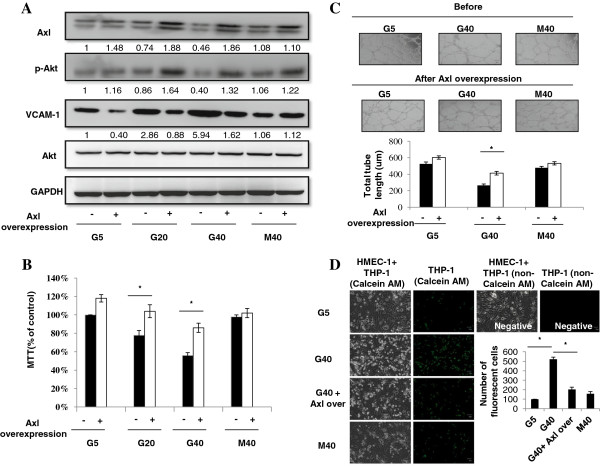
**HG concentrations and Axl transfection in HMEC-1 s.** The Axl transfection significantly reversed HG-induced Akt phosphorylation and VCAM-1 protein expression **(A)** and increased cell viability and tube formation **(B,C)** and decreased cell adhesion with monocytes **(D)**.

The next aim of the investigation was to ascertain whether overexpression of Axl activity would affect the HG-induced Akt phosphorylation, VCAM-1 expression and cell dysfunctions in HMEC-1 s. To address this issue, cells were transfected with an Axl over-expressed plasmid. Transfection results revealed that the Axl transfection significantly reversed HG-induced Akt phosphorylation and VCAM-1 protein expression (Figure 5A) and increased cell viability and tube formation (Figure 5B,C) and decreased cell adhesion with monocytes (Figure 5D). The control pcDNA3 transfection did not affect the HG-induced responses and Axl transfection also did not affect HG-induced MAP kinase phosphorylation (data not shown).

### HG concentrations and Axl knockdown in HMEC-1 s

**Figure 6:**
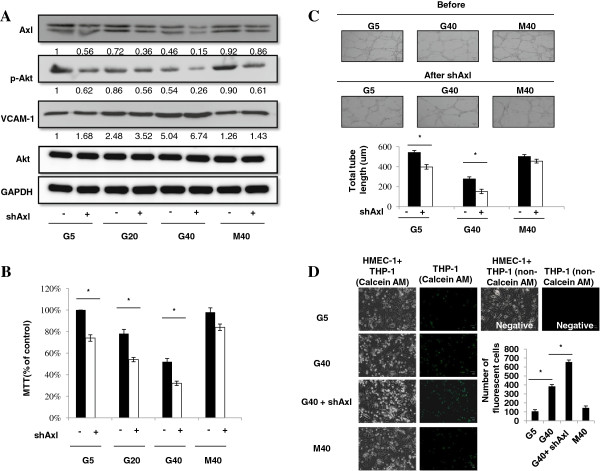
**HG concentrations and Axl knockdown in HMEC-1 s.** The HMEC-1 s were infected with Axl shRNA vectors and cultured in 5, 20 and 40 mM glucose and equimolar concentrations of mannitol. The results revealed significant additive changes in the Akt phosphorylation, VCAM-1 expression **(A)**, cell viability **(B)**, tube formation **(C)** and monocyte-EC adhesion **(D)** of Axl-shRNA-infected HMEC-1 cells in HG culture compared with HG alone condition.

In order to confirm HG-induced Akt phosphorylation, VCAM-1 expression and cell dysfunctions in HMEC-1 s through Axl signaling, experiments were carried out to assess whether Axl knockdown in HMEC-1 cells had additive effect with HG-induced Akt phosphorylation, VCAM-1 expression and cell dysfunctions. The HMEC-1 s were infected with Axl shRNA vectors and cultured in 5, 20 and 40 mM glucose and equimolar concentrations of mannitol. We detected significant additive changes in the Akt phosphorylation, VCAM-1 expression, cell viability, tube formation and monocyte-EC adhesion of Axl-shRNA-infected HMEC-1 cells in HG culture compared with HG alone condition (Figure 6A-D). The control shRNA transfection did not affect the HG-induced responses and Axl knockdown also did not affect HG-induced MAP kinase phosphorylation (data not shown). The results suggested that Axl signaling was responsible for HG-induced EC dysfunction.

### Axl modulates expression of angiogenic factors in HG-induced angiogenesis

**Figure 7:**
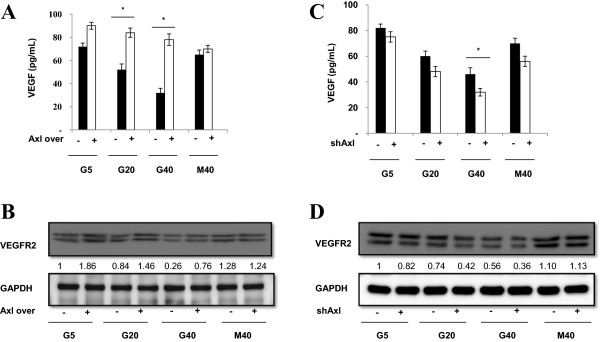
**Axl modulates expression of angiogenic factors in HG-induced angiogenesis.** The results revealed that the Axl overexpression in HMEC-1 s significantly reversed HG-induced VEGF and VEGFR2 expression **(A and B)**. The Axl-shRNA-infected HMEC-1 cells showed significant additively decreased changes in the HG (40 mM)-induced VEGF and VEGFR2 expression **(C and D)**.

Our results showed Axl was involved in HMEC-1 tube formation and HG-induced angiogenesis. We further dissected the mechanism of Axl regulation in HG-induced tube formation and angiogenesis. In the previous reports [21,23], Axl regulates processes vital for both neovascularization and tumorigenesis in tumor cell and animal models and was associated with expression of VEGF and VEGFR2 angiogenic factors. We used Axl overexpression and knockdown experiments in HMEC-1 cells to elucidate the association of Axl and angiogenic factors (VEGF and VEGFR2) expression. In Figure 7A and 7B, the results revealed that the Axl overexpression in HMEC-1 s significantly reversed HG-induced VEGF and VEGFR2 expression. Meanwhile, the Axl-shRNA-infected HMEC-1 cells showed significant additively decreased changes in the HG (40 mM)-induced VEGF and VEGFR2 expression (Figure 7C and D).

### Expression of Axl and VEGFR2 in artery from non-DM and DM patients

**Figure 8:**
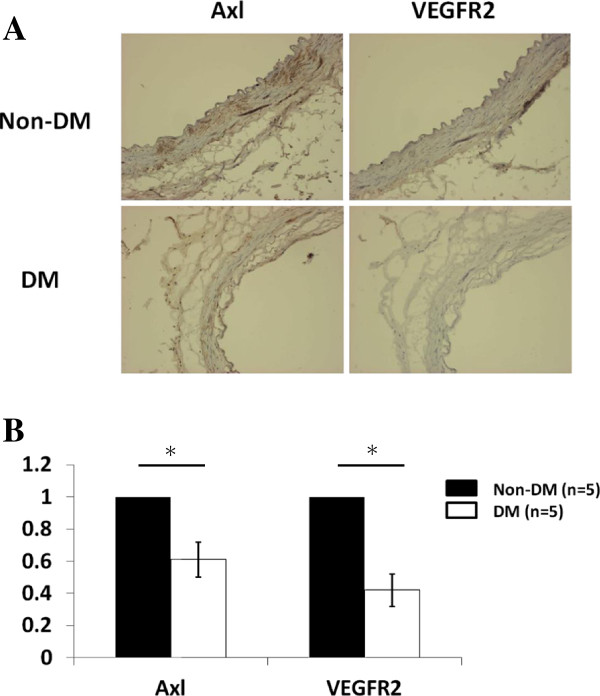
**Representative immunostaining and protein expression of Axl and VEGFR2 in DM and non-DM patients in LIMA.** DM patients showed lower expression of Axl and VEGFR2 compared with non-DM patients in immunostaining **(A)** and Western blot results **(B)** (*P* = 0.003 and *P <* 0.001, respectively).

In addition, we investigated the association between Axl and VEGFR2 expression in HG-induced endothelial dysfunction, using the left internal mammary artery (LIMA) collected from 10 patients (5 with diabetes and 5 without diabetes) undergoing elective coronary artery bypass graft (CABG). Expression of Axl and VEGFR2 in LIMA were examined by western blot and immunohistochemical staining. As summarized in Figure 8, significantly lower Axl and VEGFR2 expression were found in diabetic patients as compared with non-diabetic patients (*P* = 0.003 and *P <* 0.001, respectively). Furthermore, a significantly positive correlation between Axl and VEGFR2 expression in LIMA was noted in diabetic patients (data not shown).

## Discussion

In the present study, we dissected the role of Gas6/Axl and its downstream signaling within HG-induced endothelial dysfunction. Our results revealed for the first time that HG causes downregulation of Gas6/Axl through Akt signaling to influence the expression of adhesion molecules and VEGF/VEGFR2 in endothelial cell. These findings are supported by functional studies showing that a decrease of cell viability and angiogenesis and induction of monocyte-EC adhesion in HMEC-1 s under HG. While we manipulate the Axl expression in HG cultured HMEC-1 s, they can be through Akt signaling and modulate adhesion molecules and VEGF/VEGFR2 then regulate endothelial adhesion and angiogenic functions.

Interestingly, situations potentially associated with the vascular homeostasis either *in vitro* cell study or *in vivo* animal model, as for instance changes in pH, hydrogen peroxide, inorganic phosphate, hypertension and atherosclerosis, all seem to be affected by Axl and/or Gas6 expression but the influence were inconsistent [16,24-26]. Axl is activated by hydrogen peroxide, which is increased in vascular injury in both vascular smooth muscle cells (VSMCs) and ex vivo vessels [27]. However, recent studies have demonstrated that the inorganic phosphate-induced VSMC apoptosis and subsequent calcification are dependent on the down-regulation of the Gas6/Axl/Akt survival pathway that inhibits apoptosis and increases survival of VSMCs [28,29]. Several reports suggest a role for Axl in the pathogenesis of diabetic renal and vascular diseases. Expression of Axl and Gas6 are increased in glomerulus of diabetic rats and HG stimulation of mesangial cells, followed by induction and activation of Gas6/Axl and the Akt/mTOR pathway, which leads to mesangial and glomerular hypertrophy [15]. Cavet *et al. *[16] investigated the effects of various glucose concentrations on Axl signaling in VSMCs and demonstrated a role for glucose in altering Axl signaling through coupling to binding partners. The Axl receptor tyrosine kinase signaling system is now emerging as an important regulator of mammalian physiology and pathology with distinct and diverse mechanisms of action, depending on the cell type or organ system involved [30]. With the exception of VSMCs, there was prospective evidence linking endothelial dysfunction with atherosclerosis and demonstrating that endothelial dysfunction was the first step in the atherosclerosis. Endothelial dysfunction contributes to diabetic vascular diseases, including hypertension, atherosclerosis, and coronary heart disease, which are also characterized by insulin resistance [31]. Also, ECs cultured in HG show delayed replication, abnormal cell cycling, and increased apoptosis. However, the mechanisms by which diabetes-induced endothelial dysfunction remain poorly identified. In this study, we first confirmed plasma Gas6 molecules are inversely associated with glucose values and endothelial dysfunction markers in human. Furthermore, we used *in vitro* human EC model to elucidate HG, indeed, can result in down-regulation of Gas6/Axl signaling then cause endothelial dysfunction with decrease of cell viability and angiogenesis and induction of monocyte-EC adhesion might be through suppressing VEGF/VEGFR2 expression and activating adhesion molecules.

PI3K and Akt are downstream effectors of insulin signaling, as well as important signaling molecules in the regulation of glycogen metabolism in myocytes, lipocytes, and hepatocytes [31]. However, PI3K/Akt also plays an important role in EC functions by regulating angiogenesis, proliferation, microvascular permeability, survival and cellular transformation [32]. Recent study had shown hyperglycemia-impaired PI3K/Akt signaling may promote EC dysfunction in diabetes [33]. Previous reports have shown that Gas6 appears to regulate endothelial activation at least in part by binding its cognate tyrosine kinase receptor Axl [11]. This is not unexpected as various groups found that Gas6 exerts effects on ECs via Axl and Akt-dependent pathways [34]. In VSMC *in vitro* model, Cavet *et al.* demonstrate that glucose modulates Axl signaling via different cell signaling mechanisms. In HG, Gas6/Axl stimulation increased ERK1/2 activation whereas Gas6/Axl stimulated PI3K/Akt/mTOR in LG. Furthermore, Gas6/Axl signaling increased cell survival in LG and increased migration in HG. However, the role of Gas6/Axl and downstream signaling in the context of hyperglycemia and endothelial functions has not been explored. Our results showed evidence that hyperglycemia can cause endothelial dysfunction with down-regulation of Gas6/Axl signaling. Meanwhile, its effect was through Akt signaling, but was not involved in MAP kinase signaling. After overexpression of Axl, we can find reverse the effect of its downstream signaling and EC functions. Moreover, while using loss of function with Axl knockdown, there were further impairments to its downstream signaling and cell functions. Activation of Axl and its intracellular signaling pathways PI3K/Akt has been shown to play a crucial role in HG-induced EC viability and angiogenesis.

Diabetes is associated with abnormal angiogenesis. The effect of HG on angiogenesis is often presented as a stimulatory one, on the basis of what is observed in the late phase of diabetic retinopathy, defective angiogenesis has been observed in different models such as wound healing [35] and embryonic growth [36]. In diabetic patients and in rodent models of diabetes, collateral vessel development, in response to coronary vessel occlusion has been shown to be impaired by HG [32]. Angiogenic response to tissue ischemia via hypoxia-inducible transcription factors (HIF-1α and -2α), triggers a coordinated response of angiogenesis and arteriogenesis by inducing the expression of growth factors, such as the VEGF, the angiopoietins, and the transforming growth factor-β1 (TGF-β1). A few animal models have shown that HG affects VEGF/VEGF receptor signaling pathways, causing the arrest of vascular development with regards to ongoing physiological angiogenesis [37]. It is also becoming apparent that Gas6/Axl can further influence cell function by modulating Wnt, VEGF, and integrin-mediated signaling [22,38,39]; as such, signaling-independent mechanisms have even been suggested [40]. Previous reports revealed that cross talk between Gas6/Axl and VEGFR2 and Axl stimulation by Gas6 resulted in inhibition of the ligand-dependent activation of VEGFR2 and the consequent activation of an angiogenic program in vascular ECs [22]. Our results are in agreement with previous reports that HG reduced VEGF/VEGFR2 and Gas6/Axl expression. We demonstrated that Axl overexpression in HMEC-1 s significantly reversed HG-induced VEGF/VEGFR2 expression. Meanwhile, the Axl knockdown in HMEC-1 cells showed significant additive decreased changes in the HG-induced VEGF/VEGFR2 expression. Furthermore, we also found higher Axl and VEGFR2 expression on LIMA tissue in non-diabetic patients compared with diabetic patients. Moreover, in diabetic patients, a significant association between Axl and VEGFR2 expression in LIMA was noted. To the best of our knowledge, our results are the first to confirm that HG can influence on EC tube formation and angiogenesis by modulating Gas6/Axl and VEGF/VEGFR2 signaling.

## Conclusions

In conclusion, the results of our study demonstrate that exposure to increasing concentrations of glucose results in altering EC viability, angiogenesis and adhesion functions through Gas6/Axl/Akt signaling. If Gas6/Axl/Akt signaling defects contribute to diabetic vascular complications, then therapeutic restoration of activity may have clinical significance. We suggest that upregulation of Axl and Akt signaling pathways within the endothelium should be considered as a target for future therapeutic modalities in protecting diabetic patients from vascular complications.

## Abbreviations

DM: Diabetes mellitus; EC: Endothelial cell; Gas6: Growth arrest-specific protein 6; TAM: Tyro-3, Axl, Mer; LG: Low glucose; HG: High glucose; VEGF: Vascular endothelial growth factor; VEGFR2: Vascular endothelial growth factor receptor 2; ICAM-1: Intercellular adhesion molecule-1; VCAM-1: Vascular cell adhesion molecule-1; HMEC-1: Human microvascular endothelial cells; PVDF: Polyvinylidene fluoride; CABG: Coronary artery bypass grafting; LIMA: Left internal mammary artery; NGT: Normal glucose tolerance; IGT: Impaired glucose tolerance; OGTT: Oral glucose tolerance test; VSMCs: Vascular smooth muscle cells; TGF-β1: Transforming growth factor-β1.

## Competing interests

The authors declare that they have no competing interests.

## Authors’ contributions

Conceived and designed the experiments: CHL, YJH. Performed the experiments: CHL, YSS, CYL, YJH. Analyzed the data: CHL, YSS, FCH, CHH, YJH. Contributed reagents/materials/analysis tools: YSS, FCK, CYL, YJH. Wrote the manuscript: CHL, YJH. Overall responsibility: YJH. All authors read and approved the final manuscript.
